# Co nanoparticle hybridization with single-crystalline Bi nanowires

**DOI:** 10.1186/1556-276X-6-598

**Published:** 2011-11-21

**Authors:** Jin-Seo Noh, Min-Kyung Lee, Jinhee Ham, Wooyoung Lee

**Affiliations:** 1 Department of Materials Science and Engineering, Yonsei University, Seoul 120-749, Korea

## Abstract

Crystalline Co nanoparticles were hybridized with single-crystalline Bi nanowires simply by annealing Co-coated Bi nanowires at elevated temperatures. An initially near-amorphous Co film of 2-7 nm in thickness began to disrupt its morphology and to be locally transformed into crystallites in the early stage of annealing. The Co film became discontinuous after prolonged annealing, finally leading to isolated, crystalline Co nanoparticles of 8-27 nm in size. This process spontaneously proceeds to reduce the high surface tension and total energy of Co film. The annealing time required for Co nanoparticle formation decreased as annealing temperature increased, reflecting that this transformation occurs by the diffusional flow of Co atoms. The Co nanoparticle formation process was explained by a hole agglomeration and growth mechanism, which is similar to the model suggested by Brandon and Bradshaw, followed by the nanoparticle refinement.

## 1. Introduction

Magnetic nanoparticles have unique size effects that may provide insights into potential applications in various fields, such as ultra-high density information storage, color imaging, bioprocessing, and ferrofluids [1-3]. Specifically, cobalt (Co) nanoparticles have been a subject of intensive research because of its high magnetocrystalline anisotropy (7 × 10^6 ^erg/cm^3^) and large estimated critical size for single domains (approx. 70 nm) [4]. To synthesize Co nanoparticles with a controlled size and size distribution, various techniques have been utilized, including evaporation in an inert gas [5], chemical vapor condensation by either heating or laser-irradiating Co_2_(CO)_8 _precursor [6,7], and solution phase reduction of CoCl_2 _in stabilizing agents [8]. Although these techniques have demonstrated monodisperse arrays of Co nanoparticles with sizes down to 2 nm, elaborate temperature control and/or the use of complex chemical species are in demand, limiting their widespread use.

Metallic and semiconducting nanowires are another class of nanostructures that have attracted a great deal of interest because of their intriguing quantum properties and potential use for advanced nanodevices. Bismuth (Bi) is a semimetal widely explored for understanding physics in nanowire systems, because of its highly anisotropic Fermi surface, low carrier concentrations, small carrier effective mass [9-11], and long carrier mean-free path [12]. In particular, Bi nanowires can be good building blocks for thermoelectric applications, since good thermoelectric properties [13] of bulk Bi such as the large thermoelectric power (-50 to -100 μV/K) and small thermal conductivity (approx. 8 W/mK) have been demonstrated to be further improved in nanowire systems [14]. The quality of Bi nanowires is a critical requisite for success in both fundamental study and applications. We previously demonstrated that high-quality single-crystalline Bi nanowires could be synthesized using the unique on-film formation of nanowires (OFF-ON) method [12,15].

Hybridizing Bi nanowires with Co nanoparticles may be an interesting research topic. That is not only a combination of 0D nanoparticle and 1D nanowire, but it can also provide fundamental understanding of mutual interaction between thermoelectrics and magnetism. Recently, the thermoelectrics has sought a link to spintronics via groundbreaking works performed by several research groups worldwide. The studies on the spin-Seebeck effect [16] and magneto-Seebeck effect [17] were typical. The spin-Seebeck effect refers to power generation from a magnetic material under a temperature gradient, while the magneto-Seebeck effect concerns a change in Seebeck coefficient of a magnetic multilayer structure with insulting barrier inside, depending on the relative magnetizations. Although these works laid cornerstones for the investigation of interactions between thermoelectrics and spintronics, none of them included a thermoelectric material in their experiments. In contrast, we try to combine a magnetic material with a thermoelectric material at the nanoscale toward an eventual elucidation of the effects of magnetic nanostructures on the thermoelectric performance in this study.

The first step of this research is to establish a simple and reliable platform for incorporating Co nanoparticles into Bi nanowires. In this article, we report a simple method to synthesize Co-nanoparticles-embedding Bi nanowires, using a combination of the OFF-ON growth of Bi nanowires, sputter-deposition of a thin Co film, and post-annealing. The synthesis method of our nano-heterostructures is simpler than that of the subtle magneto-Seebeck structures and the thermoelectric performance of our heterostructures are expected to be more pronounced than that from the spin-Seebeck structures because of the use of thermoelectric material as a backbone.

## 2. Experimental details

**Figure 1:**
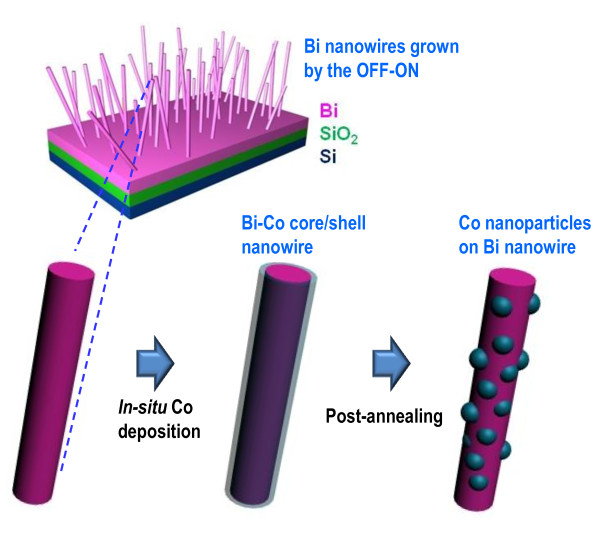
**Schematic illustration for the process of forming Co nanoparticles on the surface of Bi nanowires**. Bi nanowires are first grown by the OFF-ON method and they are subsequently coated with a thin Co film by *in situ *sputtering. Co nanoparticles are finally formed on the surface of Bi nanowires via post-annealing at elevated temperatures.

The whole process for distributing Co nanoparticles on the surface of Bi nanowires is schematically presented in Figure 1. First of all, Bi nanowires were grown on thermally oxidized Si (100) substrates using the OFF-ON method. The details of this Bi nanowire growth have previously been reported elsewhere [12,15,18]. Bi nanowires of 80-120 nm in diameter were used for this study. A very thin Co film was subsequently deposited onto the Bi nanowires by radio frequency (RF) sputtering at room temperature. This Co deposition was performed *in situ *to prevent potential surface oxidation, using the same sputtering system as for Bi nanowire growth. The thickness of Co film was varied from 2 to 7 nm by controlling the sputtering time. The as-prepared Co-coated Bi nanowires were confirmed to show Bi-Co core/shell structures with relatively uniform shell profile along the nanowire axis. The Co-coated Bi nanowires were put in a vacuum furnace for thermal annealing in the next step. The annealing temperature was controlled in the range of 200-240°C, which is below the melting point of Bi (271.3°C). The annealing time was also modulated between 1 and 5 h. A fine distribution of Co nanoparticles was obtained via optimization of the above-mentioned control parameters, as represented by the last picture of Figure 1. The evolution in the morphology and structure of the heterogeneous nanowires was investigated using scanning electron microscopy (SEM) and transmission electron microscopy (TEM). The crystallinity and composition were analyzed by the support of TEM electron diffraction patterns and energy-dispersive X-ray (EDX) spectroscopy. The effects of Co film thickness and annealing conditions on the formation of Co nanoparticles were discussed from the obtained results.

## 3. Results and discussion

**Figure 2:**
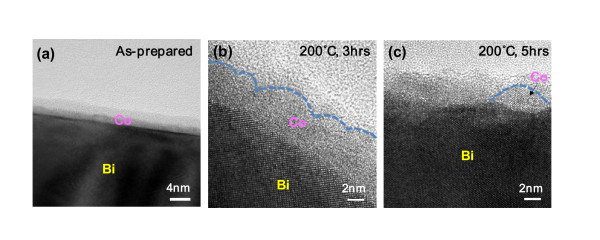
**TEM images of (a) an as-prepared and (b, c) annealed Co-coated Bi nanowires**. Annealing was performed at 200°C for **(b) **3 h and **(c) **5 h, respectively.

Figure 2 shows surface-focused TEM images of an as-prepared and annealed Bi-Co core/shell nanowires. From Figure 2a, a Co film of 3-4 nm in thickness coats a Bi core in a relatively uniform fashion and the Co/Bi interface is abrupt. Interestingly, the Co film is in general amorphous while the Bi core is single-crystalline, presumably because of the differences in crystal structures and lattice constants (Co: face-centered cubic (FCC) with *a*_0 _= 3.54 Å, Bi: rhombohedral with *a*_0 _= 4.55 Å). Once the Co-coated Bi nanowire is annealed at elevated temperatures, the Co shell begins to deform its morphology (see Figure 2b, c for morphological changes after annealing at 200°C). This is because the surface tension (1940 dynes/cm at its melting point [19]) of Co is high and Co atoms tend to relocate to reduce it under conditions where atomic motion is thermally stimulated. In addition to the intrinsic surface tension, film stress can be another driving force to induce the morphological change and it generally becomes significant at high temperatures. In this respect, additional tensile stress can be added to the surface tension of Co film at a given annealing temperature as a thermal expansion coefficient (13.0 × 10^-6^/°C) of Co is slightly smaller than that (13.4 × 10^-6^/°C) of Bi. It is found from Figure 2b, c that the morphological change is dependent on annealing time. Only multiple valleys are developed in the Co film after 3-h annealing and separate Co islands finally appear via 5-h annealing. The annealing time dependence of morphological evolution is attributed to the slowly proceeding diffusional mass flow, which is represented by the relatively low self-diffusion coefficient of Co: it is estimated to be 4.1 × 10^-32 ^cm^2^/s at 200°C using *D *= 0.37 × *e*^-67000/*RT *^cm^2^/s from [20], where *D *is the self-diffusion coefficient of Co and *R *is the gas constant.

**Figure 3:**
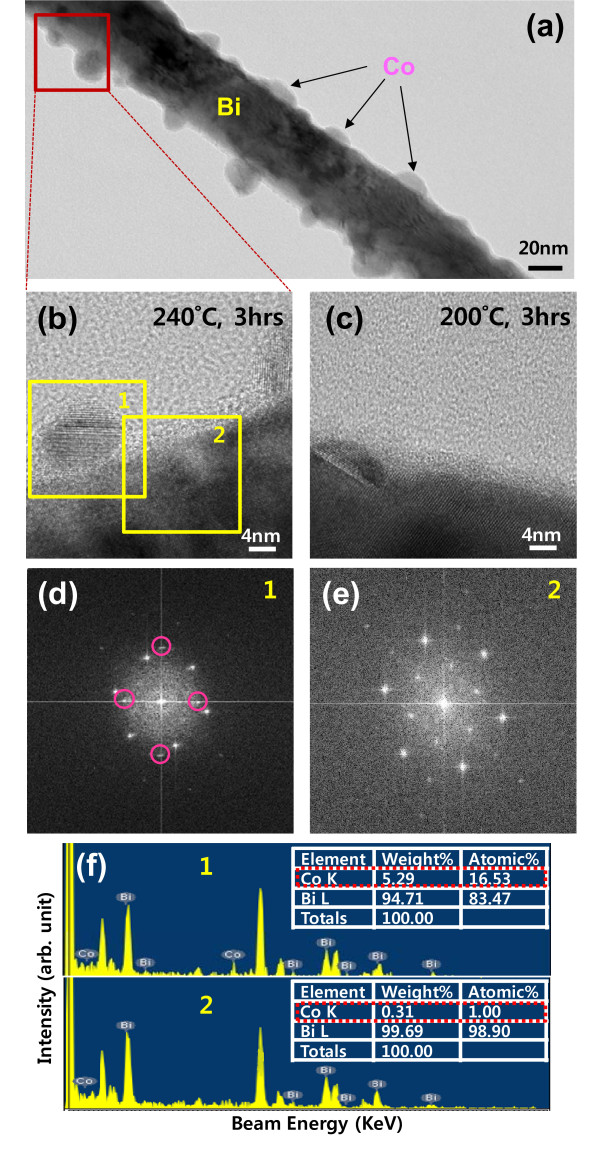
**TEM images, SAED patterns, and EDX spectra of the annealed Co-coated Bi nanowires**. **(a) **A low-resolution TEM image of a Co-coated Bi nanowire annealed at 240°C for 3 h. **(b) **A high-resolution TEM image of a selected part marked with red box in **(a)**. **(c) **A high-resolution TEM image of another Co-coated Bi nanowire annealed at 200°C for 3 h. **(d, e) **SAED patterns of the 240°C-annealed nanowire at the different areas denoted by "1" and "2", respectively, in **(b)**. The circled spots in **(d) **represent crystal planes from Co. **(f) **EDX spetra from the respective "1" and "2", showing a significant difference in Co content.

It is desirable to reduce the annealing time required for formation of Co nanoparticles. Because in this study, the Co nanoparticles are formed through solid-state diffusion of Co atoms, higher annealing temperatures accelerate the nanoparticle formation reaction following the simple Arrhenius equation. However, the annealing temperature is limited by the low melting point (271.3°C) of Bi core. Thus, it was reset at 240°C, which is the near-highest temperature where the Bi core still remains stable. Figure 3 shows low-resolution and high-resolution TEM images, selected area electron diffraction (SAED) patterns, and area-specific EDX spectra of the Co-coated Bi nanowire annealed at 240°C for 3 h. For comparison, a high-resolution TEM image of the same Bi-Co core/shell nanowire undergone annealing at 200°C for 3 h is also presented. The Co shell thickness was about 1 nm thinner than previous ones in Figure 2. Indeed, Co nanoparticles are found on the surface of Bi nanowire, as shown in Figure 3a. The nanoparticles are overall hemispherical in shape, but their sizes and inter-particle spacings are somewhat irregular, in the ranges of 8-27 and 3-32 nm, respectively. To closely examine the Co nanoparticles, high-resolution TEM was taken on the selected part of Figure 3a and its image is shown in Figure 3b. Two Co nanoparticles are approximately 16 nm distant from each other and no residual Co is observed in between them. The gray-colored layer on the surface of Bi nanowire is Bi oxide that was formed in ambient. Unlike the as-deposited Co film in Figure 2a that is almost amorphous, the Co nanoparticles look highly single-crystalline with a crystal orientation different from that of Bi core. This most likely occurs since Co atoms are ordered into a stable FCC structure through local diffusion at elevated temperatures to relieve surface tension and film stress and demonstrates the capability of our method for hybridizing high-quality nanoparticles with high-quality nanowires.

The single-crystalline Co nanoparticles are also observed from a Bi-Co core/shell nanowire annealed at 200°C for the same period of time (see Figure 3c). However, the degree of shape completion of the 200°C-formed Co nanoparticles is worse than those from 240°C annealing. Considering that a 1-nm-thicker Co film did not evolve into Co nanoparticles after annealing at 200°C for 3 h (Figure 2b), these results indicate that annealing temperature is indeed a key control parameter in nanoparticle formation. To further investigate the crystallinities and compositions of the above-mentioned Bi nanowire and Co nanoparticles, SAED and EDX analyses were performed on Co nanoparticle area (named "1") and Bi core area (named "2"), respectively. From two SAED patterns shown in Figure 3d, e, it is found that the area "1" contains extra spots (circled ones) other than characteristic Bi spots, which represent major crystal planes of FCC Co, while the area "2" shows only clear Bi spots. This indicates that the nanoparticles are really crystalline Co in accord with a TEM image in Figure 3b. The gray background of Figure 3d may come from the oxide layers on Bi core and Co nanoparticle. In addition, the EDX spectra (Figure 3f) from both areas show that significant Co peaks come out of the nanoparticles, whereas no meaningful Co peaks are observed on Bi core, reflecting that the identity of the nanoparticle is Co. The Co concentration (< 20 at.%) from "1" and non-zero concentration (0.5-1.5 at.%) from "2" are presumably caused by the limited spot size (approx. 20 nm) of electron beam.

**Figure 4:**
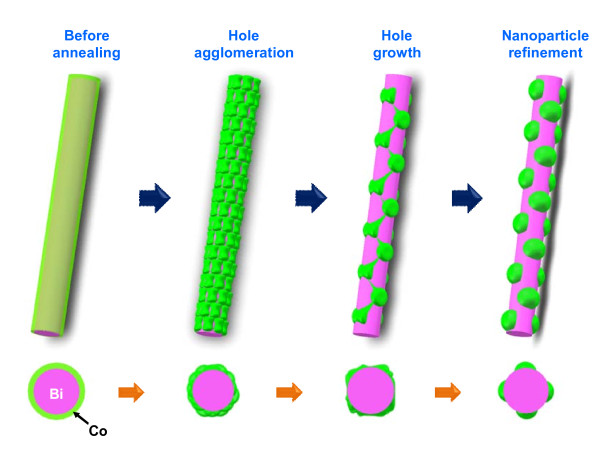
**Annealing-time-dependent morphological evolution of Co film based on our suggested mechanism**. Many vacancies in the Co film coalesce into tiny holes and the holes are again agglomerated in the early stage of annealing. Further annealing drives the holes to grow until neighboring holes encounter each other, leaving behind Co islands. Lastly, the Co islands are reshaped into near-hemispherical nanoparticles. The bottom row shows top views at the respective stages.

We speculated on the mechanism for Co nanoparticle formation. Figure 4 schematically shows our suggested mechanism, in which a reduction of surface tension, hole agglomeration and growth, and nanoparticle refinement cooperatively work. First, a near-amorphous Co thin film including a plenty of vacancies begins to modify its morphology through local atomic diffusion in the initial stage of annealing (see the second panel of Figure 4 and 2b). This process spontaneously occurs to reduce the high surface tension and film stress of the Co film. This is thermally stimulated at elevated temperatures, and mediated by vacancy coalescence, leading to local holes in the Co film [21,22]. In this initial step, local Co crystallites already start to form inside the film, as shown in Figure 2b. In the next step (longer annealing), the holes grow until neighboring holes encounter each other, pushing out Co film finally to form Co islands (see the third panel of Figure 4). According to the model of Brandon and Bradshaw, the hole radius (*R*) has a relationship with annealing time (*t*) and film thickness (*d*) as *R *= 5π^1/2^*Bt*/2*d*^3/2^, where *B *is proportional to *D*_s_γ/*T *[23,24]. Here *D*_s _and γ are the surface diffusion coefficient and surface energy of Co, and *T *is the absolute temperature. From the model, the final hole size becomes larger as the annealing time and surface energy increase and film thickness decreases, which is the case in this study. The Co islands are refined in both shape and crystal quality in the last step (see the last panel of Figure 4). More hemispherical and more crystalline Co nanoparticles come out via this step to further reduce the surface tension and volume energy of individual nanoparticles.

## 4. Conclusions

We hybridized single-crystalline Bi nanowires with crystalline Co nanoparticles, using a combination of the OFF-ON nanowire growth, thin film deposition, and post-annealing. A Co thin film coated on a Bi nanowire began to deform its morphology via thermal annealing at elevated temperatures, which is driven by the high surface tension of the film. Local valleys developed in the Co film after a short time of annealing, and Co nanoparticles finally appeared on the surface of Bi nanowire through annealing for a time longer than a critical value, leaving behind Co-free Bi surface in between them. The time required for Co nanoparticle formation was shorter at a higher annealing temperature, suggesting that this process is governed by the diffusional flow of Co atoms. Interestingly, the crystalline Co nanoparticles were obtained from an initially near-amorphous Co film using our method. The whole process of Co nanoparticle hybridization with Bi nanowire was explained by the hole agglomeration/growth and nanoparticle refinement mechanism. The hybrid nanostructure would be a good testbed for exploiting multidisciplinary nanophysics. Various nanoparticles made of materials with high surface tension could be hybridized with a variety of nanowires, employing this simple method.

## Abbreviations

Bi: bismuth; Co: Cobalt; EDX: energy dispersive X-ray spectroscopy; OFF-ON: on-film formation of nanowires; RF: radio frequency; SAED: selected area electron diffraction.

## Competing interests

The authors declare that they have no competing interests.

## Authors' contributions

JSN designed the experiment, analyzed the data, and drafted the manuscript. MKL conducted Bi nanowire growth and hybridization of Co nanoparticles with Bi nanowires. MKL and JH carried out SEM and TEM measurements. WL directed and coordinated all the experiments. All authors read and approved the final manuscript.
